# The Photographs of Meaning Program for Pediatric Palliative Caregivers and Its Impact on Meaning, Well-Being, and Perceived Social Support

**DOI:** 10.1089/pmr.2020.0046

**Published:** 2020-06-25

**Authors:** Kathryn Levy, Pei C. Grant, Rachel M. Depner, Kelly E. Tenzek, Lynda K. Beaupin, Megan E. Pailler, Erin Brewer-Spritzer

**Affiliations:** ^1^ Department of Research, Hospice & Palliative Care Buffalo, Cheektowaga, New York, USA.; ^2^ Department of Planning and Research, Trocaire College, Buffalo, New York, USA.; ^3^ Department of Counseling, School and Educational Psychology, University at Buffalo, the State University of New York, Buffalo, New York, USA.; ^4^ Department of Communication, University at Buffalo, the State University of New York, Buffalo, New York, USA.; ^5^ Department of Pediatric Hematology and Oncology, Cancer and Blood Disorders Institute, Johns Hopkins All Children's Hospital, St. Petersburg, Florida, USA.; ^6^ Department of Psychology, Roswell Park Cancer Institute, Buffalo, New York, USA.

**Keywords:** caregiver, pediatric palliative care, photographs of meaning program, photovoice, social media, well-being

## Abstract

***Background:*** Caring for a child or adolescent with palliative care needs can significantly influence the physical, mental, financial, and social well-being of caregivers. Due to this multifaceted impact, there is a demand for evidence-based support that meets the distinct needs of this population.

***Objective:*** This study aims to evaluate the effect the Photographs of Meaning Program (POM) has on meaning and purpose, well-being, and perceived social support of pediatric palliative caregivers (PPCGs).

***Design:*** Over an eight-week period, participants followed a meaning-making curriculum via social media. Following the social media portion of the intervention, a reflection and closure event and a community photograph exhibit were held. Participants completed pre- and post-test measures.

***Setting/Subjects:*** Eighteen PPCGs participated in POM. Settings included participant homes, the medical campus of the palliative care program, and a community art gallery.

***Measurements:*** Participant meaning and purpose were assessed through the Meaning in Life Questionnaire. The Flourishing Scale measured PPCG well-being, while the Social Provisions Scale assessed perceived social support. Participants were also given a satisfaction survey at study closure.

***Results:*** Presence (*p* = 0.003) and search (*p* = 0.023) for meaning were both positively impacted at post-test. Participants' ratings of well-being (*p* = 0.037), overall social support (*p* = 0.004), areas of attachment (*p* = 0.003), social integration (*p* = 0.026), reassurance of worth (*p* = 0.002), and guidance (*p* = 0.014) significantly increased from pre- to post-test.

***Conclusions:*** POM is an effective social media-based intervention for PPCGs. Future research should assess the effectiveness of this intervention in populations with differing demographics and its effect on other psychosocial indicators.

## Introduction

The high burden of caring for a child or adolescent with palliative care needs in a nonclinical capacity is well documented.^1–11^ Pediatric palliative caregivers, or PPCGs, make up 7.3% of the population^1^ and most commonly are caring for their own child due to ailments, including mental health problems, developmental disabilities, and other neurological diseases.^2^ PPCGs are significantly younger than caregivers of adults, spend more time caregiving per week,^1,2^ and describe much heavier financial burden.^1,2,4^

The responsibility of child caregiving can have a significant negative influence on the life of a PPCG, with higher burden associating with lower quality of life.^5–8^ Physical and psychological health is often compromised, with PPCGs reporting poorer health outcomes than adult caregivers.^1,4,9^ There are significantly greater levels of depression, anxiety, insomnia, and psychological distress.^2^ Finding balance and maintaining well-being can be difficult for PPCGs due to the ever-changing circumstances and limited flexibility.^8,10^ Social well-being is negatively affected when PPCGs have less time available to invest in relationships and end up feeling isolated from friends and their community.^1,4^ The impact to social well-being is especially important, as social support can be an uplifting factor for PPCGs, both mediating stress and increasing resiliency.^4,8,11^

Due to the effect of caregiving for a child or adolescent with palliative care needs, there is a call for evidence-based support interventions for PPCGs.^3^ Currently, 64% of all caregivers report that they do not receive psychosocial services despite 92% being interested in receiving support.^12^ The major barriers to utilizing support services include time and scheduling difficulties as well as guilt in leaving their care recipient. Therefore, looking at different delivery modes accessible through phones or online may help meet the psychosocial needs of these PPCGs.

The Photographs of Meaning (POM) program was developed as a more accessible modality for psychosocial intervention for populations with barriers to traditional service utilization.^13^ This intervention utilizes aspects of both meaning-centered psychotherapy and photovoice through social media platforms. Meaning-centered therapy is an individual- or group-style therapeutic intervention originally designed to enhance the quality of life for terminal cancer patients.^14–17^ More recently, meaning-centered psychotherapy has been used for caregivers of terminal cancer patients.^12,18^ Photovoice, a form of participatory action research, has been used to document and normalize the experiences of often unseen populations through photography and narratives.^19–21^ It has been widely used and often includes a photograph exhibition component to educate the greater community.

In 2017, POM was tested for feasibility within the PPCG population.^13,22^ During this iteration, participants were able to remain engaged in the social media intervention, experienced an increase in the overall presence of meaning in their lives, and expressed overall satisfaction with POM-PPCG. POM has also been utilized with adolescents and young adults (AYAs) with cancer.^23^ Participants here also expressed satisfaction with content as well as use of a social media platform, with the intervention demonstrating potential to increase the psychosocial well-being of AYAs.

Beyond the initial feasibility testing of POM-PPCG,^13^ the efficacy of this intervention on improving psychosocial factors is unclear. Evaluating the potential effectiveness of this psychosocial resource may provide evidence for more standardized availability. The aim of this study is to assess the impact POM-PPCG has on the meaning and purpose, well-being, and perceived social support of individuals acting as the primary caregiver to a sick and/or medically fragile child.

## Materials and Methods

### Study design

This is a longitudinal, mixed-methods survey study of POM-PPCG. Data were collected between June and December 2018. This project was approved by the Social and Behavioral Institutional Review Board of a midsize public university in New York on April 4, 2018.

### Participants

PPCGs were recruited through a home-based pediatric palliative care program in Western New York during June and July 2018. Inclusion criteria for the study were as follows: (1) primary pediatric caregiver (2) age 18 or older; (3) with at least one child currently enrolled in the pediatric palliative care program; and (4) access to an intervention delivery system, that is, e-mail and text messaging. PPCGs who had participated in the feasibility testing of this program were excluded from this recruitment.

A total of 27 PPCGs meeting the inclusion criteria agreed to participate and completed consent documents, with 18 participants ultimately completing POM-PPCG. Participants were compensated for their time and contribution with gift cards upon consent and after completing the exit survey.

### Measures

#### Meaning in life

The Meaning in Life Questionnaire (MLQ)^24^ was utilized to assess meaning in life. This measure consists of 10 items on a 7-point Likert scale ranging from “absolutely untrue” to “absolutely true.” Within this measure are two subscales: presence of meaning and search for meaning. Previous psychometric evaluation of the MLQ has found acceptable reliability as well as convergent and discriminant validity. This measure was utilized in the POM-PPCG feasibility study^13^ and given pre- and postintervention.

#### Subjective well-being

Participant subjective well-being was measured using the Flourishing Scale (FS).^25^ This eight-item measure also uses a 7-point Likert scale, here ranging from “strongly disagree” to “strongly agree.” This scale measures the respondents' perceived success in their relationships, purpose, self-esteem, and so on. The FS is both acceptable psychometrically and is strongly associated with other measures of psychological well-being.^26–30^ This was a pre- and postintervention measure.

#### Social support

Perceived social support was assessed using the Social Provisions Scale (SPS).^31^ In additional to overall social support, there are six subscales (Attachment, Social Integration, Reassurance of Worth, Reliable Alliance, Guidance, and Opportunity for Nurturance). The 24 items are measured on a 4-point Likert scale ranging from “strongly disagree” to “strongly agree.” Reliability and validity testing has found the SPS to be an acceptable measurement tool.^31–34^ Like the FS, this measure was also given pre- and postintervention.

#### Additional measures

Demographic information was collected from each participant at consent. Attributes included age, gender, race/ethnicity, marital status, religion, and relationship with the child receiving pediatric palliative care. In addition, participants reported on age, gender, race/ethnicity, primary diagnosis, and length of stay in the predication palliative care program of child recipients.

Participant satisfaction was measured through an *ad hoc* survey at study closure. PPCGs were asked about their overall experiences with POM, specific aspects within the intervention, and the impact POM-PPCG had on them. This included items on a 7-point Likert scale ranging from “absolutely disagree” to “absolutely agree” and open-ended questions.

### Procedures

Clinicians of the pediatric palliative care program were asked to assist by introducing POM-PPCGs during home visits and referring PPCGs who showed interest. Still, members of the research team called all eligible participants for the study. During recruitment visits, participants completed written consent, a media waiver, and all measures. During the social media portion of POM-PPCG, participants were sent via text message the weekly theme information twice each week. See Table 1 for a complete listing of themes. A modified version of the SHOWeD method questions (e.g. What do you see in your photograph? How does this relate to your life? etc.)^35^ was included in these communications to assist in creating their narrative, although PPCGs could answer weekly themes in whatever way they felt comfortable and appropriate. Using the social media app on an iOS device (in this intervention, PixStori was used), participants would choose a photograph, add typed and/or audio narrative, and upload their post to a secure portal. PPCGs were not given guidelines on photographs, allowing for greater creativity and expression. All posted content was regularly monitored by the research team to assure the safety of participants. Participants were able to view each other's posts, but no additional interaction features were enabled for PPCGs. Following the social media intervention and based on the recommendations from participants of the feasibility study, a reflection and closure event was scheduled for after the social media portion of the intervention. This event was open only to participants of the program and was designed as an opportunity to meet face-to-face and open dialogue between participants with semistructured questions. All participants were also contacted to choose their favorite photograph narrative to be printed and displayed as part of a community photograph exhibition. The two-week exhibit was displayed at a local art gallery with an opening night reception honoring the participants and included photographs, typed narrative, audio narratives accessed through QR codes, and an audiovisual reel of all additional photograph narratives. Both of these events were scheduled in advance and participants were given magnets indicating the dates, times, and locations during consent visits. Reminder phone calls about these events were also made to PPCGs. At the end of the intervention, participants completed post-test measures.

**Table 1.** Photographs of Meaning Program for Pediatric Palliative Caregiver Weekly Themes

Week	
1	Identity exploration and reflection
2	Awareness regarding sources of meaning
3	Historical sources of meaning and identity
4	Present and future sources of meaning, identity, and legacy
5	Attitudinal sources of meaning: barriers and burdens
6	Creative sources of meaning: engagement in life beyond caregiving
7	Exploration of engagement with life
8	Reflection on experience and hopes for the future

### Data analysis

All analyses were completed with IBM SPSS Statistics Version 26. Descriptive statistics, including frequencies, were conducted on demographics, community engagement, and exit survey measures. To determine if there were differences between PPCGs who participated and those who only completed the consent visit and pretest measures, independent samples *t*-tests were conducted. Q-q plots were used to determine pre- and post-test normality. Reliability of intervention measures was also explored at pre- and post-test. Paired samples *t*-tests were used to analyze the differences between participants pre- and postintervention.

## Results

### Participant demographics

The average age of PPCGs in this study was 38.06 years (standard deviation [SD] = 9.365). Participants were primarily female (88.9%), white/Caucasian (83.3%), Christian/Catholic (50%), and married (44.4%). With the exception of one participant who was the great grandmother of the child with palliative care needs, all PPCGs were parents. As some participants had more than one child in the pediatric palliative care program, there were a total of 21 care recipients among the 18 participants. On average, care recipients were 8.10 years old (SD = 6.107), had been in program for 19.44 months (SD = 14.634), and most commonly presented with a congenitally based primary diagnosis (57.1%). For complete demographic information, see Table 2. In comparing the 18 participants who completed the intervention and those who did not (*n* = 9), significant differences only emerged between preintervention scores on the SPS measure of Reassurance of Worth (*p* = 0.015). Those who did not participate had significantly higher Reassurance of Worth before the intervention than those who were active participants.

**Table 2.** Demographic Data for Caregivers and Care Recipients

Caregiver (n = 18)		
	M	SD
Age	38.06	9.365

	n	%
Gender		
Female	16	88.9
Male	2	11.1
Race		
White/Caucasian	15	83.3
Black/African American	2	11.1
Bi- or multiracial	1	5.6
Marital status		
Married	8	44.4
Single	3	16.7
Divorced	2	11.1
Long-term relationship	4	22.2
Widowed	1	5.6
Religion		
Christian/Catholic	9	50
None	5	27.8
Spiritual	3	16.7
Relationship with care recipient		
Mother	15	83.3
Father	2	11.1
Great-grandmother	1	5.6

Care recipient (n = 21)		
	M	SD
Age	8.10	6.107
Length of stay in program (in months)	19.44	14.634

	n	%
Gender		
Male	10	47.6
Female	11	52.4
Race		
White/Caucasian	14	66.7
Black/African American	5	23.8
Bi- or multiracial	2	9.5
Primary diagnosis		
Congenital	12	57.1
Neurological	8	38.1
Cancer	1	4.8

*M*, mean; SD, standard deviation.

### Reflection and closure event

Out of the 18 participants, only 2 (11%) were able to attend the reflection and closure event. One of the two participants also brought along another PPCG who was not taking part in the intervention. Participants appreciated meeting face-to-face after seeing each other's posts and expressed they wished they could meet the others who could not attend. They also welcomed the opportunity to connect not only the shared experience of caregiving but also in the discussion of shared health care providers within the community. The varying diagnoses of their care recipients did not appear to affect discussion.

### Community photograph exhibit

Twelve POM-PPCG participants were able to attend the opening of the community photograph exhibit with their families, in addition to a large number of community members. Community members were asked to complete a brief survey on their experience at the exhibit. From the 60 surveys returned, the primary reasons for attending the event included “to support the [participants] honorees” (37%) and “to learn” (35%). While 45% of attendees felt they had a high level of knowledge on caring for a sick/medically fragile child, 68% felt the exhibit changed their perspectives on the lives of PPCGs and 97% would recommend the exhibit to others. The full catalog of the framed artwork from the exhibit can be found in Supplementary Table S1.

### Pre- and postintervention measures

Reliability testing of the MLQ subscales found that internal consistency ranged from 0.867 to 0.902 at pre- and post-testing, which is acceptable. There was a significant increase in Presence of Meaning for PPCGs, with scores increasing by 2.53 points on average (*p* = 0.003). Conversely, Search for Meaning scores dropped significantly by 4.58 on average after participating in POM-PPCG (*p* = 0.023). Refer to Table 3 for the complete statistical findings for all intervention measures.

**Table 3.** Mean Differences Between Pretest and Post-test Measures (*n* = 18)

Measure	Subscale	Pretest	Post-test	t	p
		M (SD)	M (SD)		
MLQ	Presence of meaning	27.94 (4.815)	30.47 (4.125)	−3.426	0.003^b^
	Search for meaning	22.29 (8.447)	17.71 (7.346)	2.506	0.023^a^
FS		46.59 (6.295)	48.24 (4.790)	−2.280	0.037^a^
SPS		81.76 (9.731)	86.76 (7.989)	−3.350	0.004^b^
	Attachment	13.59 (2.002)	14.82 (1.667)	−3.441	0.003^b^
	Social integration	12.41 (2.033)	13.47 (1.375)	−2.447	0.026^a^
	Reassurance of worth	12.47 (1.807)	13.59 (1.543)	−3.631	0.002^b^
	Reliable alliance	14.76 (1.921)	15.12 (1.317)	−0.899	0.382
	Guidance	14.00 (2.424)	14.76 (2.107)	−2.748	0.014^a^
	Opportunity for nurturance	14.53 (1.546)	15.00 (1.581)	−1.411	0.177

^a^ Significant at 0.05.

^b^ Significant at 0.01.

FS, Flourishing Scale; MLQ, Meaning in Life Questionnaire; SPS, Social Provisions Scale.

Internal consistency for the FS was *α* = 0.816 at pretest and *α* = 0.835 at post-test, both of which are acceptable. There was significant increase in scores by 1.65 points following the completion of POM-PPCG (*p* = 0.037). This suggests an overall increase in the subjective well-being of participants after taking part in the intervention.

Reliability for all subscales of the SPS at pre- and post-test met the acceptable levels of internal consistency. POM-PPCG participants experienced a significant increase in their perceived social support after completing the intervention, with an average overall increase of 5 points (*p* = 0.004). Statistically significant changes occurred within the Attachment (*p* = 0.003), Social Integration (*p* = 0.026), Reassurance of Worth (*p* = 0.002), and Guidance (*p* = 0.014). No significant changes occurred within the Reliable Alliance or Opportunity for Nurturance subscales.

### Satisfaction

The response from participants after completing POM-PPCG was overall very positive. PPCGs found the format of the social media intervention effective, as it made it easy to participate (100% mostly or absolutely agree), and the weekly themes helped them better understand their experiences (93.8% mostly or absolutely agree). For those who were able to attend the community photograph exhibit, 69.2%, it was an important and meaningful part of the intervention experience. Participants mostly or absolutely felt that after completing POM-PPCG, they had more meaning in their lives (68.8%), were more satisfied with their social support (37.5%), and felt a greater sense of connection (62.6%). In general, 100% of participants feel they benefited in some way and 100% would recommend POM-PPCG to other PPCGs. A summary of the satisfaction survey data can be found in Table 4.

**Table 4.** Summary of Participant Satisfaction Survey Responses

	Neither agree nor disagree (%)	Somewhat agree (%)	Mostly agree (%)	Absolutely agree (%)
The social media aspect of POM-PPCG made it easy for me to participate			25	75
The weekly themes helped me make sense of my experience		6.3	50	43.8
The community photograph exhibit was an important experience for me	30.8^a^		15.4	53.8
After completing POM-PPCG, I feel that I have more meaning in my life	6.3	25	43.8	25
After completing POM-PPCG, I am more satisfied with the social support in my life	18.8	43.8	12.5	25
After completing POM-PPCG, I feel I have a greater sense of connection with something greater than myself	12.5	25	18.8	43.8
Overall, I feel that I benefited from participating in POM-PPCG			31.3	68.8
I would recommend this program to others caring for a seriously ill child/adolescent			25	75
Additional comments:				
“This was a wonderful experience. I'm so glad we participated. I very much enjoyed the opportunity to pause and reflect on the experience we've had throughout the journey.”				
“Overall I really enjoyed participating in POM-PPCG. It gave me a formal platform to think about my role in my son's life as a caregiver, father, and husband.”				
“I really feel POM-PPCG gave me the opportunity to discuss everything that was important to me.”				

^a^ These PPCGs were unable to attend the community photograph exhibit.

POM-PPCG, Photographs of Meaning program for pediatric palliative caregiver.

## Discussion

The study assessed the relationship between participation in POM-PPCG and participant meaning and purpose, well-being (flourishing), and perceived social support. In terms of both presence and search for meaning, participants experienced significant changes. Individuals with high presence of meaning and lower search for meaning are generally satisfied with their understanding of purpose and are aware of what makes their lives meaningful.^24^ Results of the FS exhibited a small but significant change to participant well-being. The greatest increases occurred on items involving meaning, purpose, and optimism about the future, echoing the changes in the MLQ. The majority of participants in this study did not see themselves as caregivers, but simply as being a parent. Being a parent in itself, while complex, is highly associated with meaning in life as well as satisfaction.^36,37^ POM-PPCG may help those caring for a sick child better recognize the satisfaction stemming from this role. Many facets of perceived social support increased for participants after completing POM-PPCG, with the greatest occurring in PPCG attachment and reassurance of worth. While caregivers of sick children often find themselves physically isolated due to the hours of committed care, POM-PPCG enhanced social support without increasing proximity. This comes from both the benefit of the intervention and the understood motivation to share via social media. Individuals are driven to sharing information through social media for many reasons, including interest in forming a community,^38,39^ social connectivity,^40,41^ and acquiring or providing empathic support for others.^42,43^ Combining these motivations with targeted intervention creates the means for psychosocial advancement.

Despite efforts made to promote presence throughout the intervention, attendance was lacking at both of the in-person events, with only 11% of participants attending the reflection and closure event and 69% attending the community photograph exhibit. Anecdotally, participants reported that scheduling issues and transportation difficulties were the main factors that hindered attendance. For caregivers in general, time, scheduling difficulties, and feelings of guilt often hinder their ability to take part in psychosocial services.^12^ Furthermore, alternative delivery methods of therapy or counseling are noted as their preferred method of care. This information, partnered with the low attendance of events and high POM-PPCG engagement, demonstrates the importance of appropriate intervention delivery based on intended participants. This goes beyond the current intervention as well as the PPCG population; there are a number of individuals who could benefit from psychosocial services, but traditional delivery methods may impede access. Consideration of how services can be delivered based on population deserves additional scrutiny. In terms of POM-PPCG, the social media intervention is the most important component. However, incorporating ways for participants to interact virtually may further enhance the experience in situations when attending in-person events is not feasible.

### Limitations

This study has some limitations that must be considered. Although the sample was more diverse than that of the feasibility study,^13^ it consisted primarily of white female mothers. Future studies should aim for greater diversity to better assess the generalizability of POM-PPCG. Sample size and subsequent quantitative findings must also be considered with caution. Furthermore, this intervention had two components that gave optional physical attendance (reflection and closure event and photograph exhibit). While both events were reported to be very valuable by the attending participants, there were attendance issues due to constraints. Future studies should consider adapting the curriculum to have easier access for participants. Third, this study was able to provide iOS devices on an as-needed basis to participants. As this may not always be possible, future studies should consider the most feasible ways to reach participants through social media for successful intervention availability and delivery. Finally, future iterations of POM-PPCG should aim to use additional follow-up to determine if the postintervention changes are maintained over time.

## Conclusion

POM-PPCG is a successful social media-based intervention for individuals providing care to sick and/or medically fragile children in a nonclinical capacity. For this study, nearly all PPCGs identified as the parent of the child care recipient. Participant meaning in life, well-being, and perceived social support were all positively affected by the intervention and 100% of participants would recommend its use for other PPCGs. Low attendance of participants at in-person components further solidifies the need to adapt therapeutic efforts for greater accessibility. Additional efforts should be made to test the effectiveness of POM-PPCG in populations with differing demographics and its impact on other psychosocial indicators.

## Supplementary Material

Supplemental data

## Data Availability

In accordance with the Social and Behavioral Research Institutional Review Board approval of this project, all data including audio recordings and transcripts are to be kept secure, private, and not to be shared.

## Abbreviations Used

FS: Flourishing Scale
*M*: mean
MLQ: Meaning in Life Questionnaire
POM-PPCG: Photographs of Meaning Program for pediatric palliative caregiver
SD: standard deviation
SPS: Social Provisions Scale
