# Supplementary material for: The Photographs of Meaning Program for Pediatric Palliative Caregivers and Its Impact on Meaning, Well-Being, and Perceived Social Support
Source: Palliat Med Rep. 2020 Jun 25;1(1):84–91. doi: 10.1089/pmr.2020.0046 (PMC8241342; doi:10.1089/pmr.2020.0046)
Supplement: Supplemental data [file Supp_TableS1.pdf]

Supplementary Data

Supplementary Table S1. Complete Catalog of the Photographs of Meaning Program for Pediatric Palliative Caregiver Community Photograph Exhibition[a]

Photograph	Complete narrative
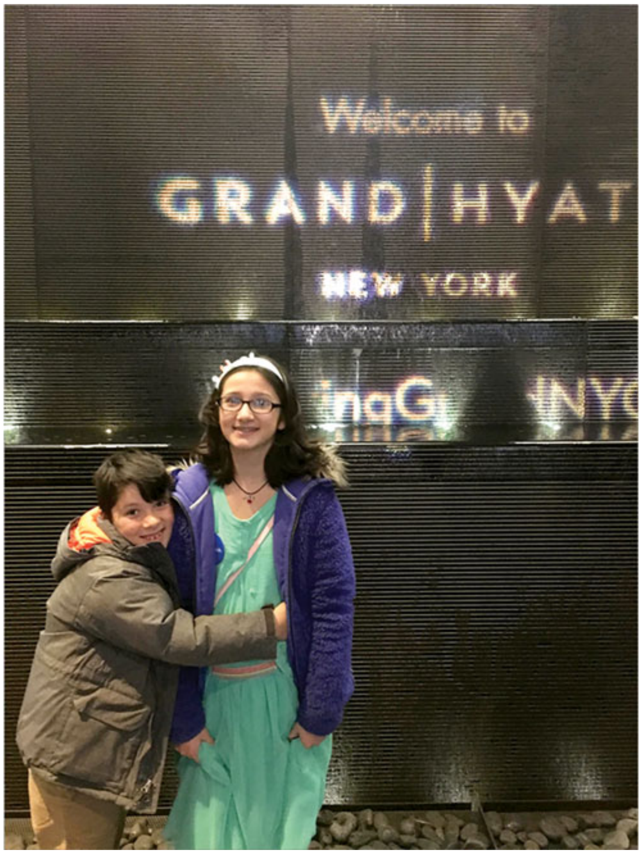	Better late than never is my week one post about what is meaningful to me. This picture obviously represents my two children and they are my reason for everything, and I… am remembering here, my daughter's a Make-A-Wish recipient and so this was our family trip to New York City for her wish that was granted and it just brings me back to that moment of happiness and togetherness and hope and joy, so, um… it was a very meaningful week for us in New York City as well, especially for her, and one that will, that continues to last with her and gives her strength and courage and um, hope.
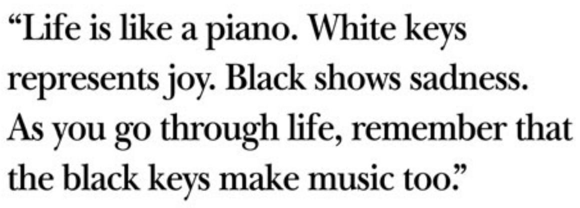	I thought I'd introduce myself with um, I don't know, an inspirational quote that helps put everything into perspective. When you have a good day, when you have a bad day, when you have just an okay day. Um, it's good to have some perspective.

(*continued*)

Supplementary Table S1. (Continued)

Photograph	Complete narrative
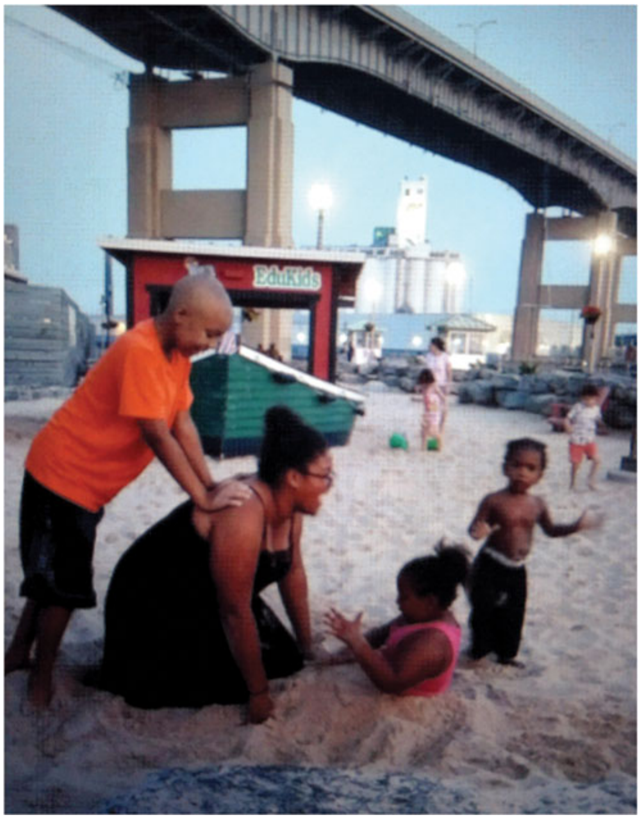	Seeing my kids happy is what is really meaningful to me. It's important because I love to make memories with them.
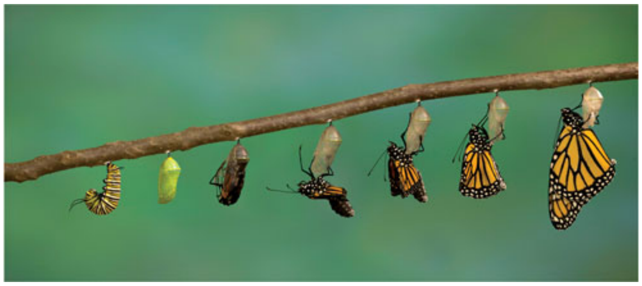	So to be completely transparent, this was definitely a little bit harder than I thought. And then I finally decided on this picture of the process of the butterfly because before I had my daughter, little caterpillar just hanging out doing his thing loving life, and things are great, and then he goes through this process where he's alone and he's dark and he's ugly and then he goes through this process of then becoming this beautiful butterfly. And I don't know, it just kind of popped into my head and resonated with me, because the process of having a sick child, it's definitely a process, it's always ongoing, but I have to say before I had my daughter obviously I was very carefree and didn't really look at things the way that I look at things now. Clearly she's not all grown, she's eleven. But now I look at things, the last eleven years have been very rough but they've absolutely changed me in a better way and I can't imagine my life any other way. However there's been some very dark times and there's been some very amazing times. But as I look back and reflect, I wouldn't want my life any other way. I also wouldn't want to wish my life on anyone else as well. But I truly look at having her as a beautiful gift, and, I don't know, the pictures of the process of a caterpillar to a butterfly I feel just completely represents the whole process of having, for me having all the issues with my daughter, her ongoing medical issues, and beyond her medical issues, just her struggles in general. But not only do I look at the process as beautiful, but I have this beautiful young girl who looks at life so, so differently than most children and this, again, I wouldn't want it for anybody else but I can't imagine my life any other way. So that's all I have to say about that.

(*continued*)

Supplementary Table S1. (Continued)

Photograph	Complete narrative
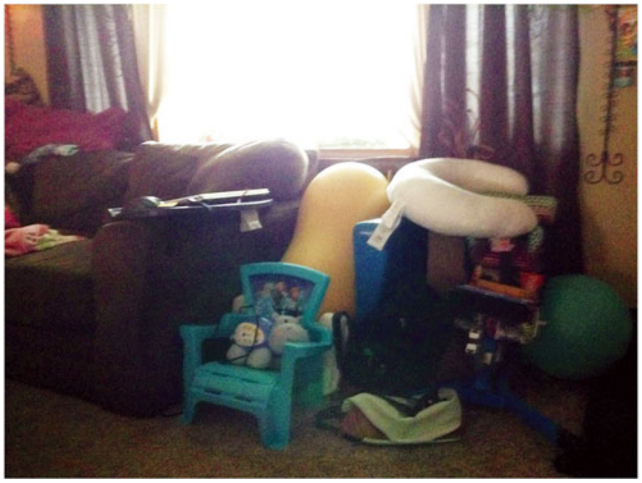	Hello everyone! It is my first time posting and I'm a little behind but life gets crazy. This photo might not make any sense to you but there are two things I am extremely thankful for in this photo. The first is all of that equipment. My daughter is very complicated and a lot of that stuff in that photo is used to help her just to reach her best potential. All of our therapists come and they're absolutely wonderful and help us with her to use that. I'm just extremely thankful for technology, um, keeping my little girl as good as she can be and reaching her best potential. The second thing in that photo is my laptop sitting on the edge of my couch. And I'm sure most of you can relate, it is very hard financially, um, to work and be a special needs mom. Therapy is eight times a week and then doctor's appointments and like today, I had to refill prescriptions and oxygen supplies and blood glucose monitors and I was on the phone with specialists getting everything situated. It took hours and that laptop allows me to work from home and still be able to provide for my family. My boss is terrific, terrifically wonderful to me and my family and I stay up very late at night sometimes working but I am very blessed that I am still able to work and take care of everybody and everything around here. We would have a much different lifestyle if I didn't work and I'm very thankful for it. So there's my first post! I'm always looking for time away from all these people that live here with me so I can do this. And I know my second post is going to be, so um, you'll see it in a minute. Thanks everyone!
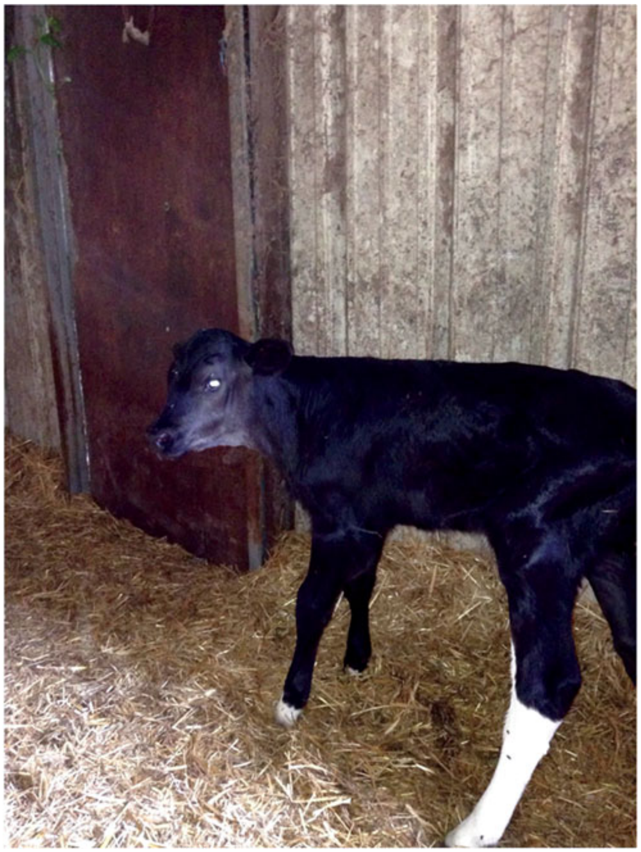	Hi, this is the farmer mom. This photograph is important to me because we have calves year round. I don't know, where we live we have about 400 cows all together so I don't know how many calves we have every year. But, a bunch, and this one happens to be a bull, so he won't be staying long, we only keep the heifer calves, um, but it is always meaningful to me because it's new life, um, you put a lot of work and effort into, you know, the cows and it's always nice when there's a new baby.

(*continued*)

Supplementary Table S1. (Continued)

Photograph	Complete narrative
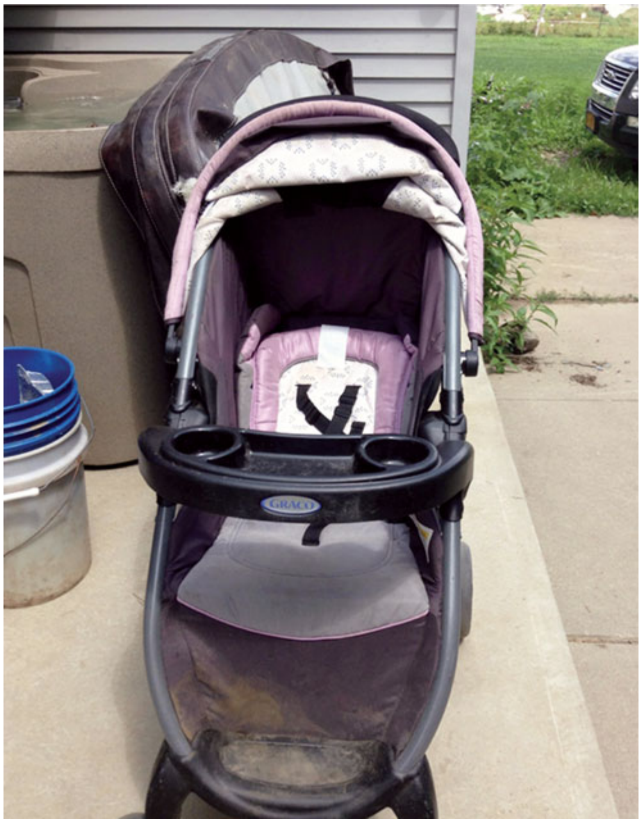	Hi, it's the farmer mom. This picture is of my granddaughter's stroller and it is meaningful to me because I have been trying to get myself healthy and walk, I've been walking everyday now. And this morning I took my granddaughter for a 3.8 mile walk. Um, it helps, it helps clear my mind, it helps me to feel better emotionally and physically and um, hopefully I can stick with that for a while, if not forever.
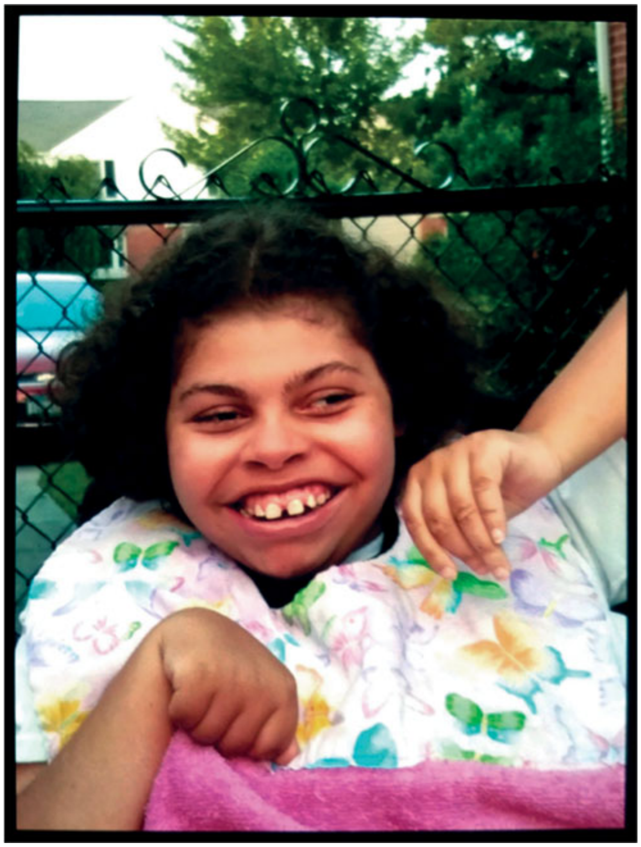	Hi everyone. This is our beautiful daughter. She's 15 years old. We're looking forward to getting to know you.

(*continued*)

Supplementary Table S1. (Continued)

Photograph	Complete narrative
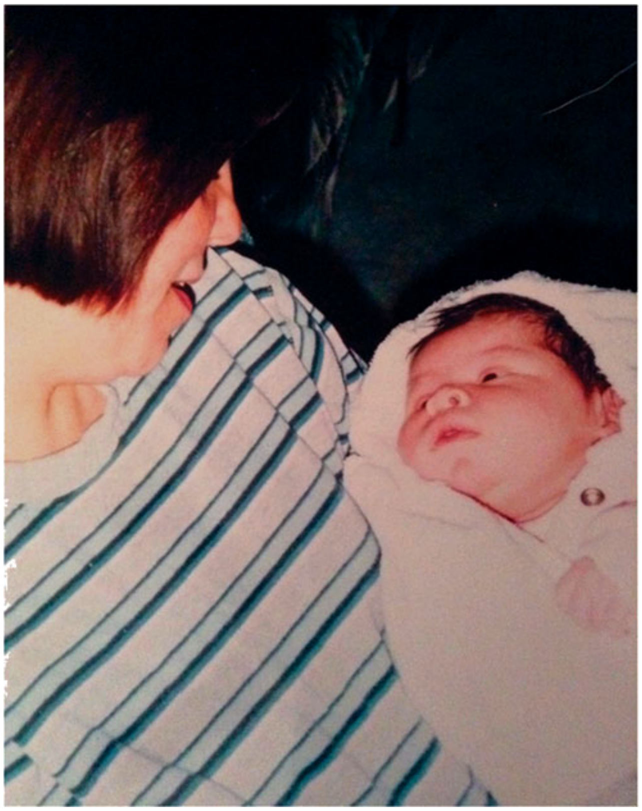	One of my favorite memories to relive is when our beautiful daughter came into our lives through adoption. I was told I shouldn't have any more children because I had some health issues and we always wanted at least one more child to add to our family. So we decided to pursue adoption and we went through the training and it took a few months, we went through the training and got approved and in July of 2002. And the time from we got approved, we had gotten a few calls and they just never worked out for whatever reason. And then in March, we got the call that would change our lives. We got a call telling us about a birth mother who was having probably a girl and she was due in about 2 weeks and would we be interested in being profiled. You made a profile of your family, you put pictures, you put different things you're interested in and so the birth mother/birth family gets to know these families. And they would go through these profiles to choose the family for their child. So we were asked if we wanted to be profiled, we said yes. And we waited probably a couple days, got the phone call, "she chose you," she would like you to be the family for her child. But they said she's not due for 2 weeks, so we thought "okay, this is great, we have time." That was a Wednesday. And Friday morning at eight-thirty, we got a phone call- "Guess what? Your girl is here. She was born overnight." So, oh my gosh, 2 days later she's here. And we had nothing. I had been looking for things over this time so I had an idea what I wanted but we didn't know if it'd be a boy or a girl. So my husband and I, so excited, first called family "She's here! She's here!" And we were supposed to pick her up two days later at the adoption agency. So he and I, my son was already at school, my husband and I went shopping and bought all the big things that we needed. It was the most exciting shopping trip we had. So on this Sunday, we went to the adoption agency and this beautiful girl was placed in our arms. This is a picture of- this is back at home, but this is a picture of my daughter and I that first, maybe the next day. But oh my gosh, we were so in love and she just made our family complete and we've been in love ever since. I was in love with her the second I knew about her. Before I even knew we were going to get her for sure. She just made our family complete, and we've all been in love, our family, our extended family. In the beginning, my son was never going out with his friends. He wanted to just stay home. He was twelve and he always wanted a sibling, so this was so exciting to him. Either his friends would come over so they could see her, and he could show her off, his beautiful sister. Or he just stayed home to spend time with her, it was really special. And as you know, I have shared he's passed away so you know those memories mean a lot, I'll share a couple pictures with my husband and son and our whole family. Those are one of my favorite memories to relive. And I tell my daughter around her birthday every year, she seems to understand, she smiles, you know. One of my favorite memories.
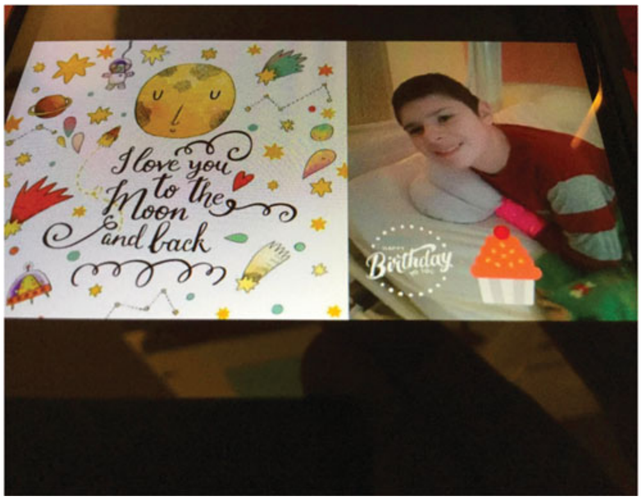	This is week 7 and the week is for love, beauty, and humor. This one pictures sums all of it up for me. Mr. Boo endures chronic pain, yet he shows all three-love, beauty, and humor. He'll blow kisses to me and I'll blow some back and then we say, "keeping all your love and kisses in my heart forever"- and he puts his hands across his heart. Beauty is within your soul. Anthony-alias, Mr. Boo-his beauty shines through all the time with his smile, his love for his family, friends and pets. He has a humorous personality. He loves to joke, say silly things, trick his family and friends. He has a gut-busting giggle and even on a very trying day he can get any one of us to laugh along with his silliness. So he is love, beauty, and humor.

(*continued*)

Supplementary Table S1. (Continued)

Photograph	Complete narrative
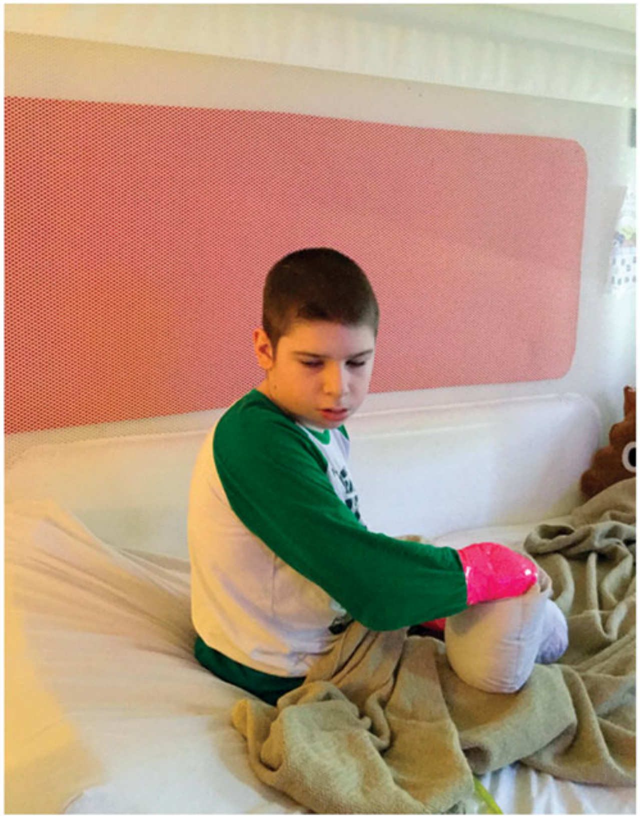	Hi, this is a picture of my boy, Mr. Boo-one of his many nicknames. He's my shining star.
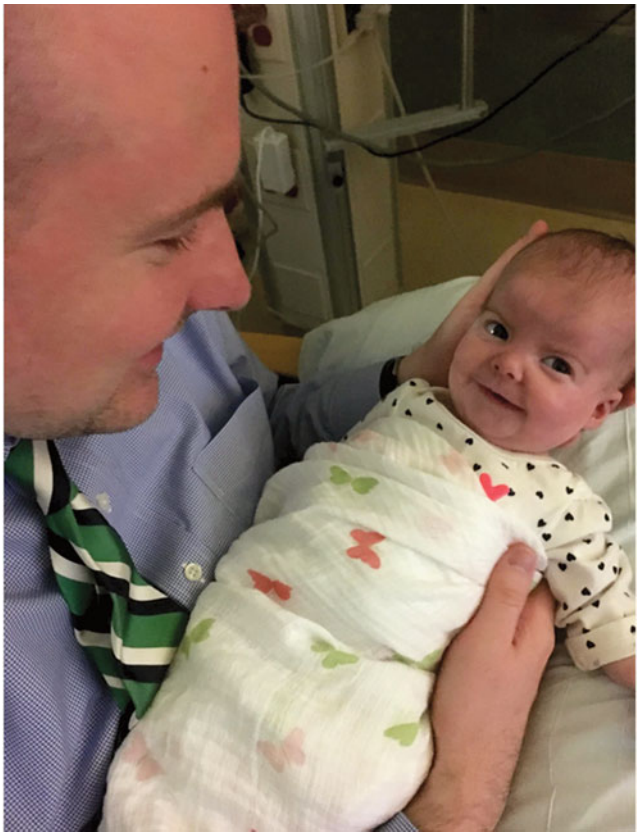	I was looking through my thousands of photos trying to think about what's most meaningful to me. And I came across this one and I remember this day distinctly because we had spent most of our daughter's life in the hospital at this point. She was only 3 months old. And, you know, everything was a question. We didn't know what the next day was going to bring. Um, this is maybe one of the first times that we saw her smiling a lot? And it was just pure joy. There was nothing better than seeing her smile and seeing her happy and it honestly made everything we had gone through up to that point worth it. I think this photo kind of shows how excited we are, you know, are and were at that point to be parents. And this is my husband holding her, and you can see how excited he is to see her happy. I think that's still incredibly meaningful for us, to know that she's happy, in however much time we have with her, that we try to make her time here as happy and as meaningful and as loving as possible.

(*continued*)

Supplementary Table S1. (Continued)

Photograph	Complete narrative
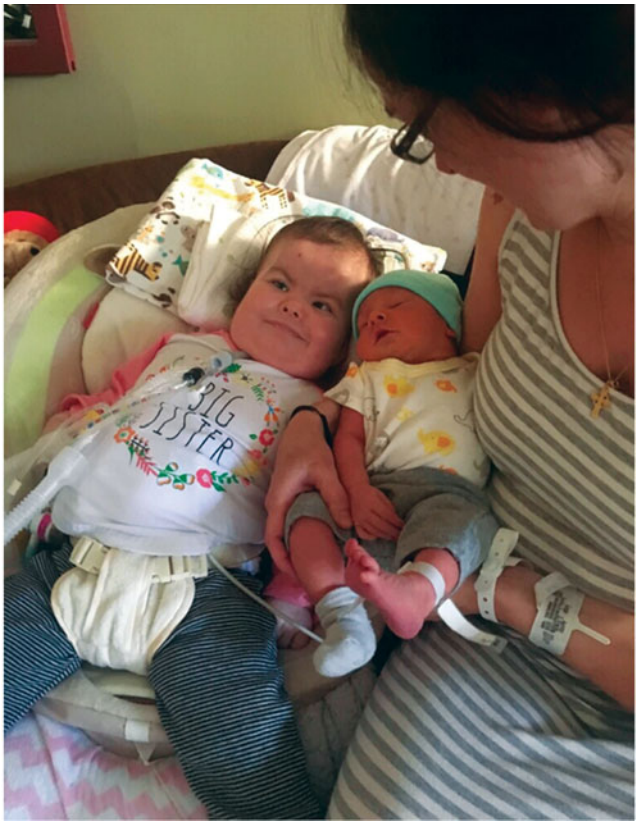	I guess if I were asked to give advice to any other family who was faced with a serious medical condition or any other kind of serious diagnosis, I think I would say that you know the love that you have for your child is still going to be as, of course, as strong as ever. And it is still going to be such an incredible experience with your child, I mean; I don't think that is any different. I think there is such a, I mean, so much joy in this experience and it um, probably sounds crazy to anyone on the outside, who thinks we must have such a hard life and um, being in and out of the hospital, and dealing with such a difficult medical condition but a lot of stuff I think is the same. A lot of other families that have healthier or typical children they deal with struggles too and ours have of course a lot different, but you just, like I said there's just so much joy in it and the love is just so strong and I mean it is just something you can't explain to anyone that doesn't have a child themselves and this whole experience is something you definitely can't explain to anyone who doesn't have a sick child but I think I would just reassure people that this is definitely a rollercoaster but um in our experience, people we meet along the way and the people that love our daughter and the people that have really cared about us it's just been amazing. I guess I chose this photo of our daughter meeting her baby brother for the first time (pauses), it's hard for me to look at without getting emotional but um, you know this wasn't expected, she, we, weren't told to expect for her to live past a few months and the fact that she um, got to meet her baby brother, and we like to believe has lived a pretty good life in her two and a half years and that is something we are very proud of, it's beyond our wildest dreams, so we are very grateful.
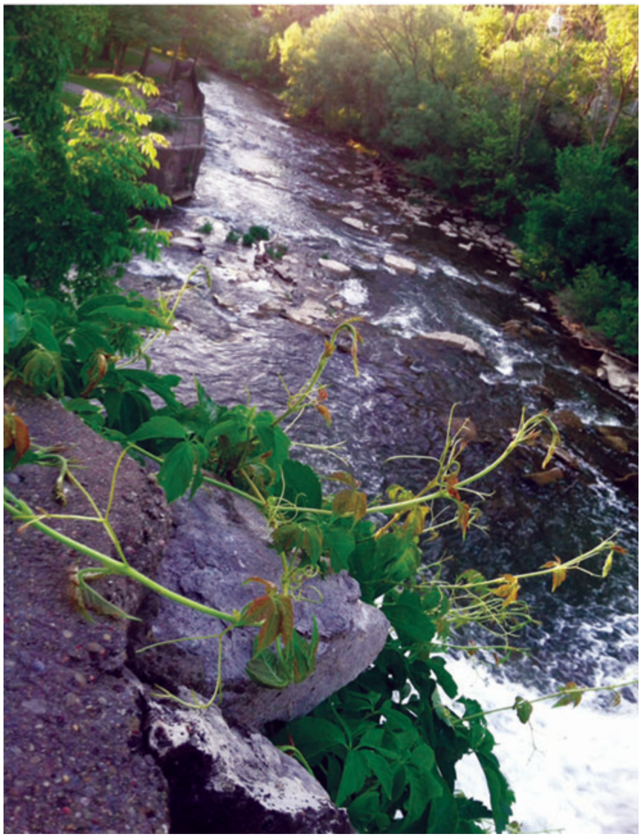	Before this adventure started with my daughter, I went wherever the wind took me and never cared about time. Now time actually matters and I'm more controlled by a clock and I hate that aspect of life right now.

(*continued*)

Supplementary Table S1. (Continued)

Photograph	Complete narrative
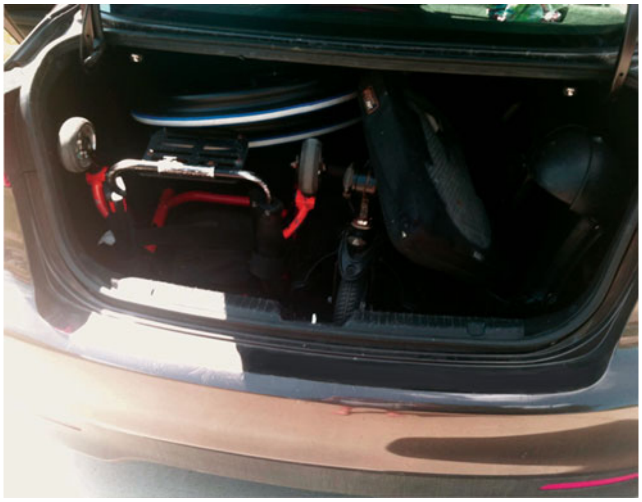	This is a picture of our trunk with my wheelchair and my wife’s scooter in the back. Our daughter’s stroller goes in the front seat and everyone’s told us to get a minivan but there’s more than one way to do things and when there’s a will there’s a way.
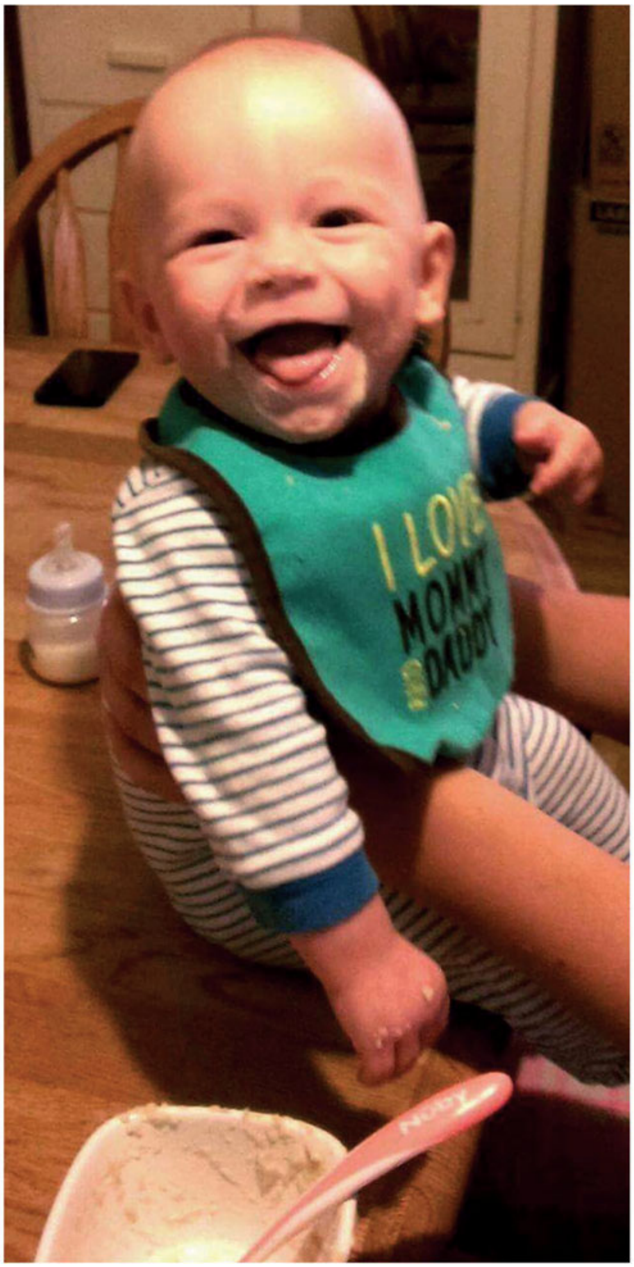	This is week number 8 and this is my other post for “hopes for the future.” In this photo this is my son when he was only a couple months old and he seemed to be one of the happiest babies. We had no idea what his diagnosis was ‘til he was about 2 years old so we were not in the loop or anything in this photo. But anyways, um, my hopes for him for the future are to be successful in life, to, um, to go to college, to find a good job, to find someone that he loves…same thing that somebody loves him and somebody takes care of him. I just hope, um, he doesn’t have bumps and everything. In his life it’s always hard to, um, take care of them and understand these types of things that we have in life but um, I just hope that he’s gonna be healthy and happy and um, just a wonderful, loving boy. I hope he…no matter if something goes bad in his life I hope I still see that happy, smiley face on him. And, um, that’s it.

(*continued*)

Supplementary Table S1. (Continued)

Photograph	Complete narrative
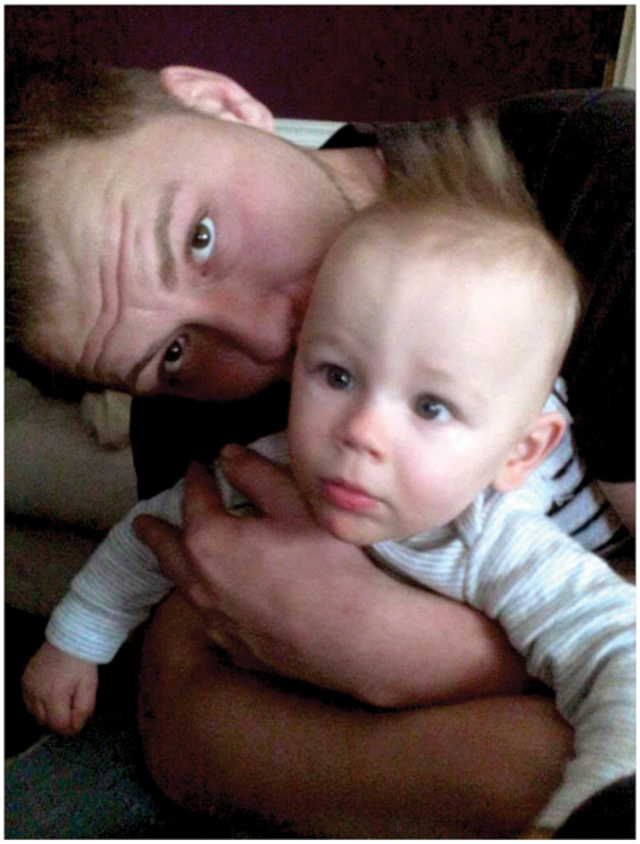	Hello, now this is week number 8 and we need to offer some advice. And honestly what I would, what I would be offering to someone with a child that is, that has a disability, or even a child that has a normal life, I would say that no matter what goes on in your child's life, make it the best for them because I was always told that when they're young, that's when they absorb everything. What you teach them, what song you sing to them, they absorb that. I also would like to say that there will always be obstacles and no matter which way it goes, if the obstacle is going downhill or the obstacle is actually really easy, no matter how you look at the obstacle you just need to try your best especially for your child no matter what the obstacle is. Of course there are things we will come across in life and it will be really difficult, it will be very heart-heavy, there's just so many things out there that we can run into and sometimes our minds and our bodies just don't know how to act. And honestly I always try and make it to be, try to make it happy, especially like for my children because I know that they're always watching, they'll always be there, always be by my side. And this goes for anyone that has kids. I just wanna let you guys know that no matter what happens in your child's life or in your life or in a loved one's life, no matter what happens, just try and make the best of it because we only have one life and we should definitely be living our life to the fullest. Now I chose this picture because the man in this picture is my fiancé and also my children's father. He is a very kind-hearted man that I can go to for anything, um, even asking like "Oh does my hair look good like this? Is this an okay shirt to wear or not an okay shirt to wear?" He's definitely someone that can give advice no matter if he's in a good mood, in a bad mood, had a bad day at work. He's definitely just somebody that I have always looked to, to go to for advice because we've been together for almost eight years. And, honestly, like, I've never lived by myself. I was living with my parents and then when him and I got together I lived with him and his parents and the a couple years later down the road we found our own place and he's always helped me through everything. Whatever obstacles I had. And once we had our first son, that was when I needed the most advice because a lot of the times I would feel so overwhelmed that I would just go to him and cry and just tell him, like, "I really feel like I'm a bad mom." And he would always be there to hold me and to let me cry on his shoulder and just, um, he would give me advice and let me know, like, that I'm not a bad mom. Just overwhelmed, I'm overworked, or I'm worked up. And I just need to go over what has happened and try to change what the circumstance was so, um, that's it.
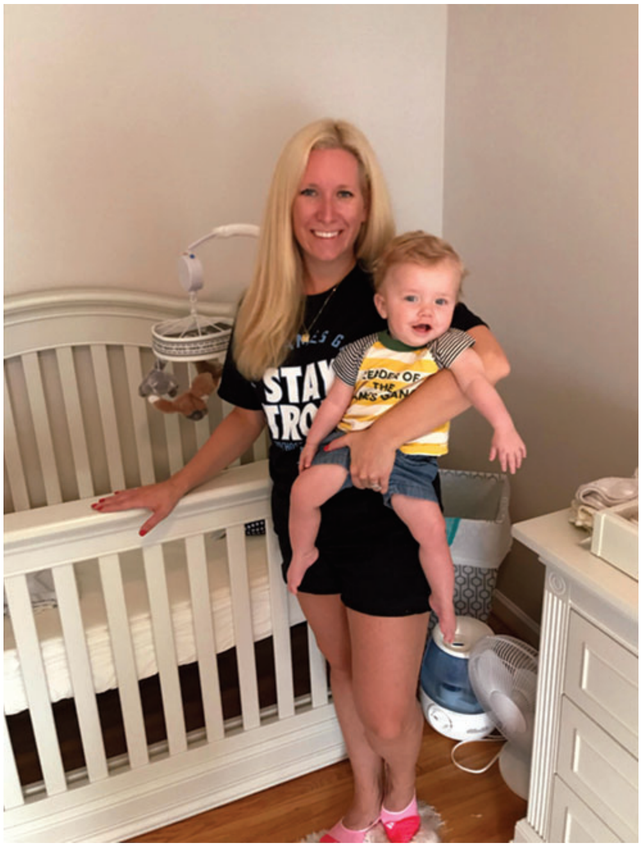	So in week 4 we had two themes, one of the themes being to create a post about a role or accomplishment, um, or something we're really proud of. So in this post, um, there's nothing for me that I'm currently more proud of than an event that we participated in this past Saturday. So we participated in our very first Muscle Walk for the Muscular Dystrophy Association. We created a team for my son. Here you can see him, he's wearing his shirt that says "Leader of the James Gang." Um, and I'm really proud of our involvement with this because as I mentioned it was our first year participating. When we got his diagnosis I sort of dove head first into "How can I get involved in things?" "How can I help?" "What can I do?" and I stumbled across this Muscle Walk so I put together a team and that was also the way that we shared our story with the community and our friends and family. We kind of put it out there on social media, said this is what's going on, if you want to get involved or help or learn more, we're doing this event, please join us, et cetera. So I'm really proud of this for a number of reasons. One of which being that we currently are the top fundraising team nationally, which is incredible. It's our first year participating and that just speaks volumes about the support and love that we are getting back from our friends and family and community and it's really helping drive us in a positive direction as we kind of go on this path of our new life, and his diagnosis, and figuring everything out. Um, I'm also really proud of myself, of the role I've taken on as an advocate, as an advocate for James, um, for the entire muscular dystrophy community, um, for all sorts of different things. This experience and, um, his diagnosis has given me a lot of new perspective and it's opened my heart to a lot of things that, you know, maybe I just wasn't tuned into before and I'm really proud of the strength that I find in advocacy for him and for other people. So, you know, when asked to think about creating a post that represents, uh, something we're proud of, an accomplishment, a role, this is perfect. I am proud to be James' mom. I'm proud to be his advocate. And I just couldn't be, I guess, more proud of where we are in this journey and where I believe we are going to go.

(*continued*)

Supplementary Table S1. (Continued)

Photograph	Complete narrative
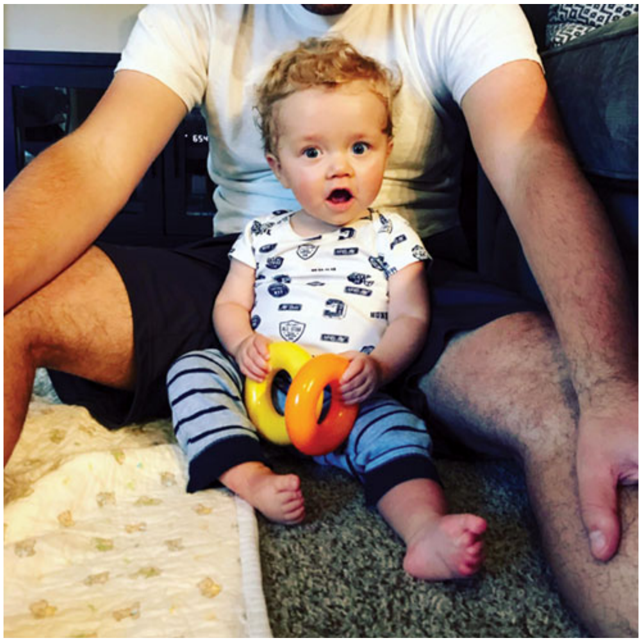	On our final post one of the two themes, um, is to create a post that shows our hopes for the future. I chose this particular picture because when I look at it, I see everything that our son is. He is sweet and curious and has great hair and is charming and funny and engaging. So when I think about the future I just think about him continuing to develop into this really sweet, smart, curious little boy. To me, this picture when I look at it I can't help but smile because it just captures him so well. So my hopes to the future are not unlike many other parents I don't think. I hope that he grows up and is happy. I want him to be happy; I never want him to be sad or not safe or unsure. And you know naturally things will come up throughout our journey as they will for any child. My job as his mom is to help him through those things and to remove the barriers and to make his life as full and happy and without limitations as possible and that's exactly what I'm gonna do. I hope that for my son when, you know, people see him down the road and he's in a wheelchair or has different types of adaptive devices or different assistance, I hope that people can see beyond that and I hope that they can see, um, see him and talk to him and get to know him. And I know already even at only 10 months old- I know that he has a beautiful personality. He already lights up so many people's lives and um, I know he will continue to do that and to be a joy for so many. So my hopes for the future for him is that he's happy, that people can see past, um, certain things-certain physical limitations he might have. I hope that he is a big brother someday. I hope the world for him. The sky's the limit and I'll do everything I can to give him the very best life he can have.
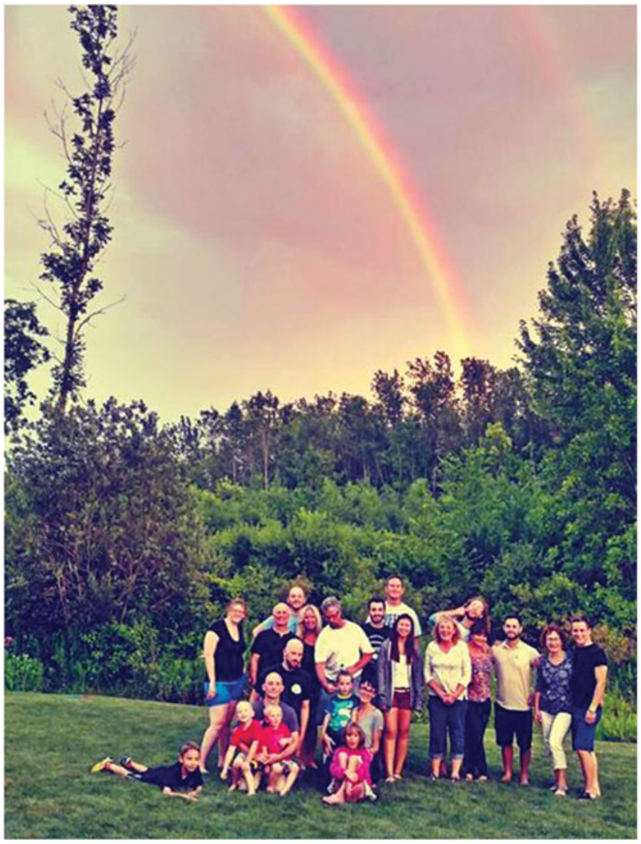	This photo shows some members of my family at a family party we had, uh, this past summer, one that was put on to celebrate the life of my late Nonna. It's become a bit of a family tradition, we call it "Nonna Fest" and, um, it's one of my favorite parties of the year because it allows, uh, me and my family to come together and spend time with each other and catch up and, um, do all the great things that, um, we love doing. Um, it's become one of the, again, one of my fondest traditions and just overall a great memory. And when I think about how this shapes me, these are really the people that have played, um, some of the most important roles in shaping who I've become and how I've grown up and, um, and have all surrounded me with, uh, a positive network of people and, um, that's obviously influenced me today.

(*continued*)

Photograph	Complete narrative
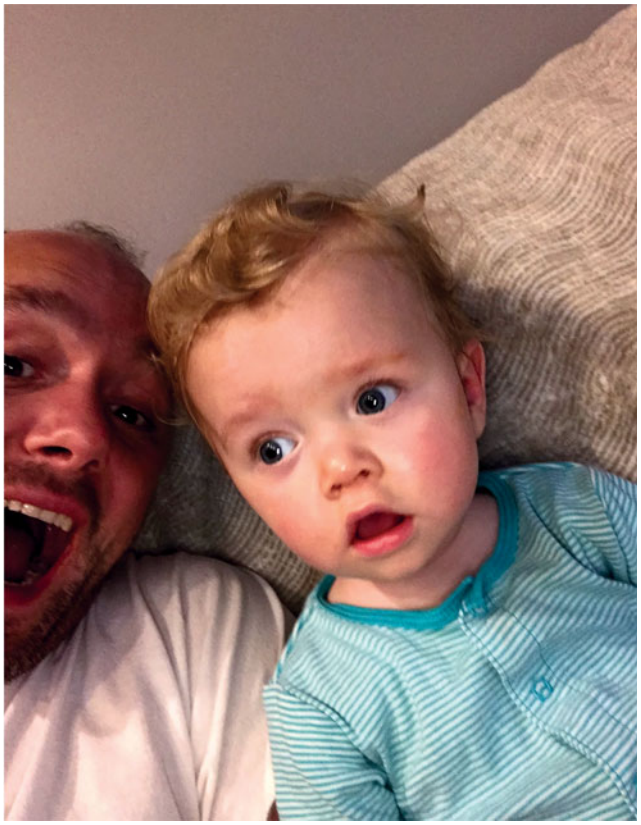	There are a lot of things in my life and accomplishments that I've made that I'm very proud of, uh, but the one I'm most proud of is being a father to my son.
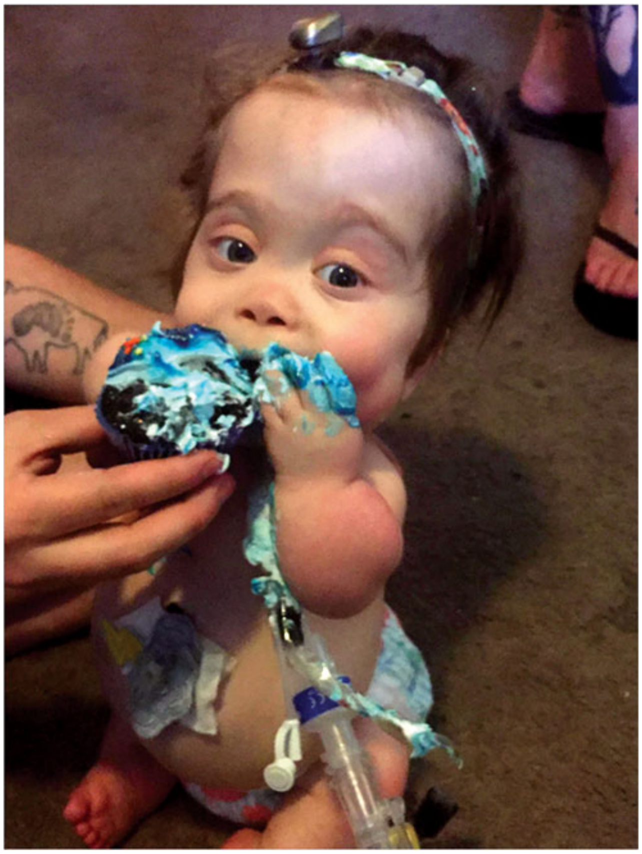	We are sharing our story about my daughter which is she has a rare type of dwarfism called Hypochondrogenesis which stems off of the type of dwarfism that I have, which is Spondyloepiphyseal dysplasia. She has just turned 4 years old and we are taking it one day at a time and we hope that you guys enjoy our journey.

(*continued*)

Supplementary Table S1. (Continued)

Photograph	Complete narrative
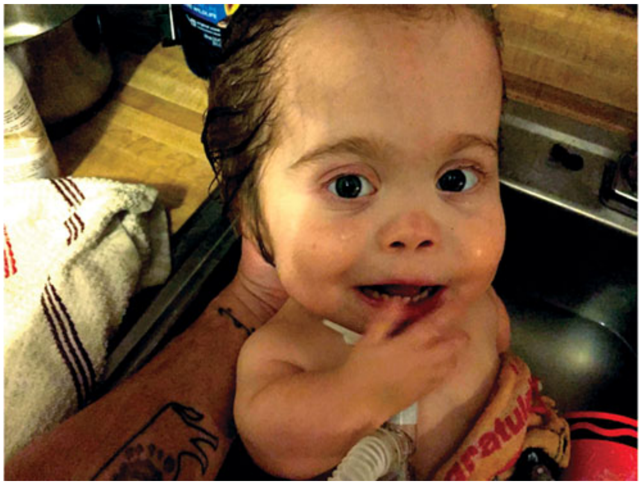	My best advice for anybody new to the medical scene and having a special needs child would have to be patience and not having a time limit or a time frame on anything. At least for my daughter, she does what she wants to do when she wants to do it. While doctors and other medical professionals say she should be doing this or should not being doing that, she is doing it and there's no rush to push them. There's no rush to find out the end results because then you miss along the way all the special, happy times. There's always ups and downs in anybody's life and especially being a medical mom, medical dad, and you just learn to really roll with things and love your child for who they are no matter what the situation is.
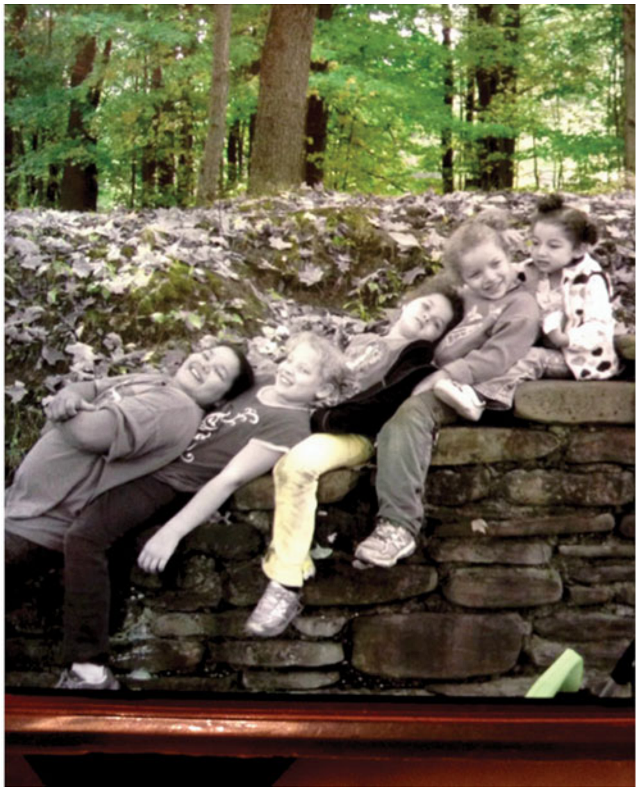	POM memories week three, meaningful memories, relationships, traditions. This one right here, this is our yearly camping trip picture of my ladies. Um, we freakin' love camping, okay? We do it once a year, we camp for a week, and we look forward to it every year, it's our only vacation. Now it's been two years we haven't gone, well one year we haven't gone- last year, and this year we're not going to be able to go. That really puts a damper on everyone. In this photograph, this is a rock wall in Letchworth State Park, I had the girls sit up on it and take all sorts of funny pictures on it. They didn't want to touch each other, you know, that kind of thing. It's the best, we have the best time camping, we enjoy nature, the animals in nature, we just love it all so we really have a good time here. We've never had a bad time, it's something that means more to me than just "we went camping" because the year their father left us, we were supposed to have our second time camping and then my daughter was born… how did this work out. We camped our first time I was pregnant with the twins, which is the kids that are right before my sick little girl. Then we skipped a year and we were supposed to camp that next year and he, my children's father, my husband, left us when I was pregnant with my daughter, and we had already had the vacation planned so that year my mother took one of the twins and the two older girls and my grandmother and they camped. And I was home with the newborn and the other twin. So I was so like "damn it, I didn't get to go camping" but when we decided we would do it again, we planned for, we were getting closer I was thinking "oh my god, how am I going to do this?" Like -unable to decipher- before but now I was like "how am I going to do this, we don't have a man! How are we going to start a fire and do wood and blah blah blah" and that was like ten years ago, okay? We've gone every year except last year and this year like I said, just us females. The first few years, us females along with my great-grandmother was a hoot, the eight of us, females in a cabin, hahahaha. But then it was just empowering, ha, we don't need a man for anything, which ends up being really good to teach your daughters, you know? So camping will always go down as a great memory for us ladies. It brings us to our, like, roots, almost. Like we can just be bare and not have to do dollop or get ready or fight over the bathroom, it's just fun, you know. I am glad we continued on and did it, so that we learned we could do everything and it's like, one of those super good feelings where you're just like I don't know what it's called but you're pissed you didn't do it earlier. But I'm so grateful to have had these little girls experience that with me. They didn't see it, that their mom was worried about how everything was going to go down. They don't see that. They just see that mom makes everything happen and it's all good. One day I will share with them how anxious I was about camping and not knowing if we would do it well enough, and now I'm a pro, not really. So that's my picture for this moment. Bye y'all.

(*continued*)

Supplementary Table S1. (Continued)

Photograph	Complete narrative
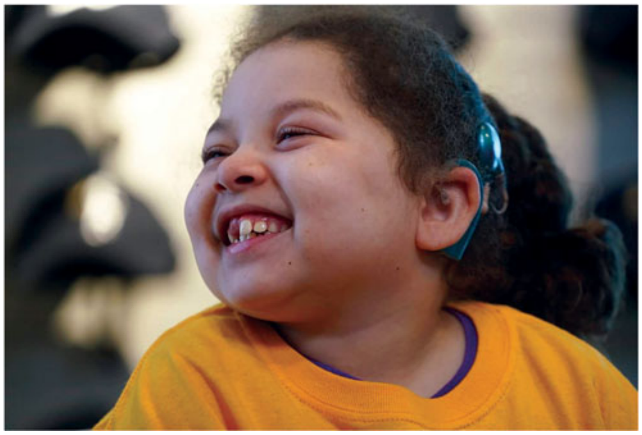	After my daughter was born, life changed drastically. She was born ill and hasn't gotten any better since. For myself, how I changed…I feel like I'm a different- I don't feel like I'm a different person but I have different agendas now. Everything is very spur-of-the-moment so even with your best laid plans you have to put in time there for error. My daughter has a lot of accessories-parts of her that need to be managed and you just have to learn every one of them. So before when your biggest nursing job was taking a sliver out of a finger or toe, now you're changing a G-tube or you're giving shots, you know, that's life changing right there for sure.
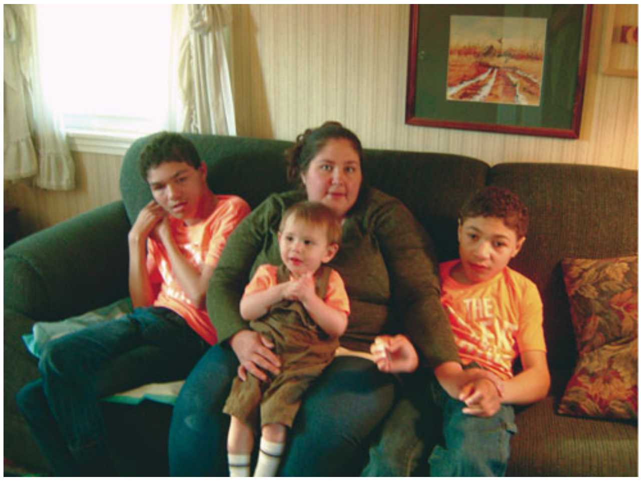	This picture is actually my second post from what was supposed to be last week. So please bear with me, I can't help losing time, especially with three special needs kids. So after becoming a caregiver, we got my first son into early intervention therapy and education in the home. So I had people in and out of my house on a daily basis, it was crazy. I was also pregnant with my second son by this time and I was wondering would he also have the same diagnosis as his brother. He was born and he did. So for fifteen years I took care of both my sons, made sure they got all their therapy and education sessions. I took them to so many doctors' appointments. I did my best and that's all I could do. Well fifteen years later, I met my now-fiancé and fell in love and we got pregnant with our third son, who also has the same diagnosis. Finding that out was a little easier for me because I was more aware of everything that could possibly happen, but he, my fiancé was new at all of this this, even at just being a parent. But he's doing a great job, I'm so proud of him and us. Thank you.
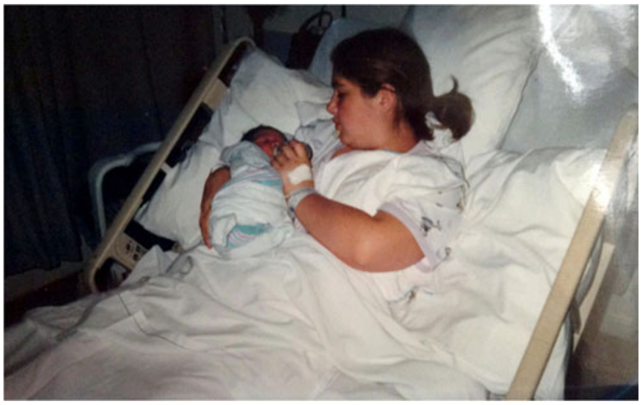	This was a proud day for me because it was the day I became a mom. It was a tough and long labor, but after twelve hours, and an emergency C-section, our son came and he was perfect. Thank you.

(*continued*)

Supplementary Table S1. (Continued)

Photograph	Complete narrative
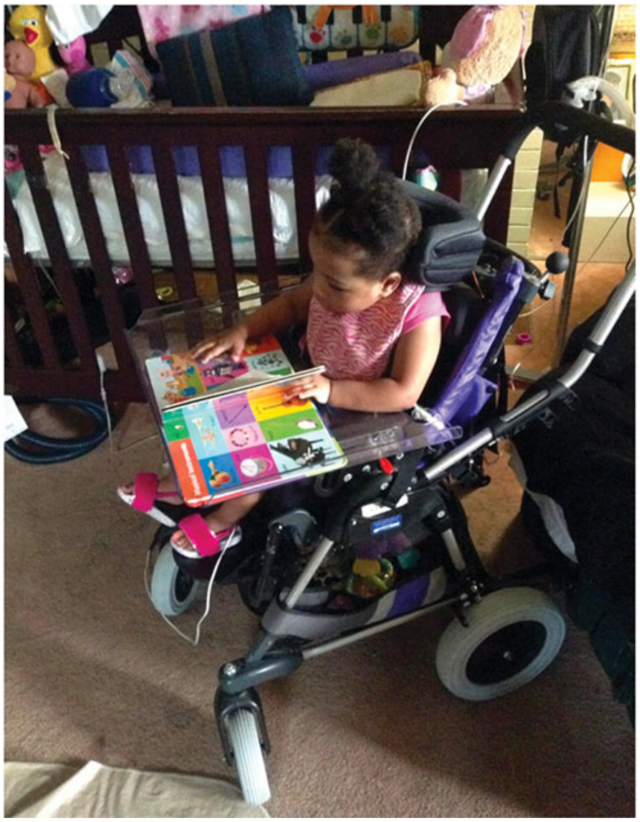	Hi everyone. Every day my daughter has activities and this particular day the activity that she wanted was to look sat her book. Um, I also posted this because this is for moving forward-the last week in the POM program. My experience with being a caregiver-the mother of this beautiful, smart, intelligent little girl-um, has been a rollercoaster. Ups, downs, happiness, sad-emotions everywhere. But through it all, I still manage to smile and move forward because at the end of the day she is a human being. She has feelings and she's motivated and determined to do what she wants to do. Um, this will affect me in the future on many different levels. It'll affect me moving forward because I know that I am capable and that I know that we all will be successful in life, including my daughter. Her siblings-my two boys-are awesome with her. And moving forward it's going to affect them because now she's the only girl and they have someone to look after- the youngest one and the oldest one. Um, I'm actually excited to have hopes for the future. And I wouldn't even say so much as hopes because my hoping is praying. I pray that we will overcome and live a happy, normal life. And she's the best thing could have ever happened to us and with this experience we've learned a lot. We've learned how to take care of not just her but other people with special needs. If I were to see someone out and they're going through-you know-or having issues, I am willing and I will go over there to help them and assist them. Um, so this is my experience and I am looking forward to doing great things with my daughter along with her siblings.
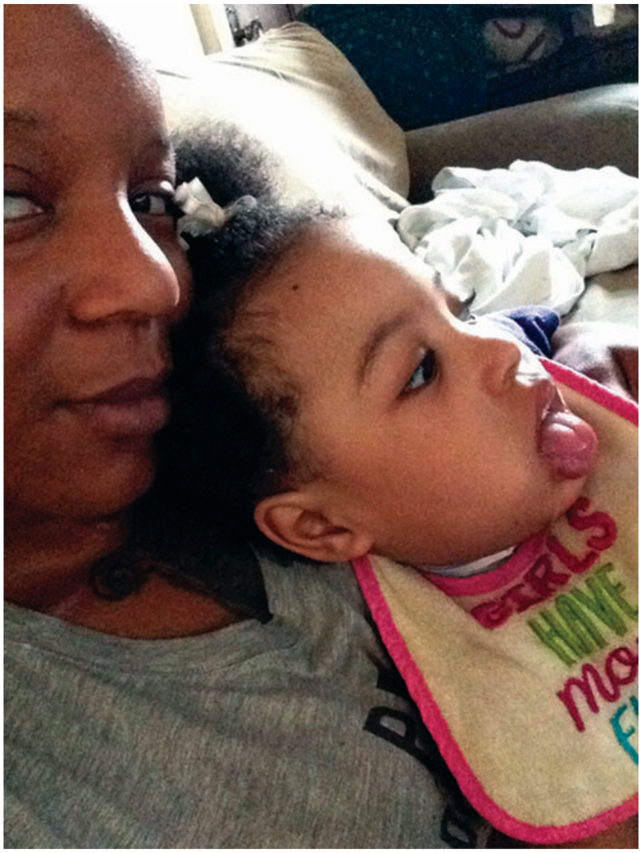	This is all about me and my identity. And who I was prior to becoming a caregiver, um, I was a single mother of one. So technically I was still a caregiver but not of a child with special needs. But that's who I was before I became a caregiver, um, I was still a single mom raising a boy, a baby boy. I was kind of lost because I had him at a young age. I found out I was pregnant when I was 18, I had him when I was 19. I graduated high school five months pregnant with him. I was somewhat on the right track but I felt like I could have done more. Everything changed about me once I had him. Things I did, I worked like crazy, more than two jobs. I started to lose friends because my responsibilities changed. My characteristics changed in a lot of ways. Um, I started to carry myself better. I started to speak differently, with respect. You know kids are like sponges. They pick up on everything. Um, it was a lot. It was a lot. And then I got into relationships that never lasted and I kind of figured at one point that I would not have any more children. So I was kind of down and out and still continuing to take of this young man here now. So yeah that's who I was before I became a caregiver for my daughter. Um, who I became after is a very strong, emotional mother. Not only did my characteristics change, body image, the things I do, the people I know—my beliefs changed in a lot of ways. I started to believe more that there is a God. Not to say that I never believed, but my faith became stronger. I never prayed so hard, even with my oldest son, I've never prayed as hard as I've prayed with my daughter. Every time she goes to the hospital, we're praying. And praying for her makes me pray more for my other two children. So when I pray for her, I pray for everyone. I'm more spiritual. Um, I'm more in tune with myself and my motherly abilities. I'm more active, I'm more hands-on. Anything that I became after becoming a caregiver has been nothing but positive vibes. And that's my motto: positive vibes. If people are being negative, thinking negative, talking negative—I can't be around it. I pick up off of vibes. Good vibes, bad vibes, I'm all for positive vibes. So with that being said, that was all about me before and after I became a caregiver. And I will always be a caregiver no matter what happens.

(*continued*)

Supplementary Table S1. (Continued)

Photograph	Complete narrative
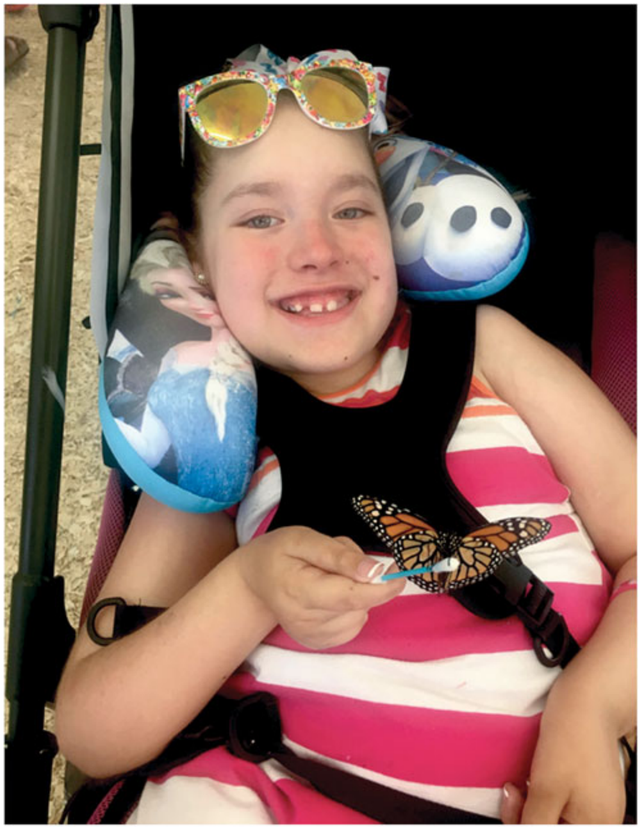	A life lesson I have learned is that my daughter's smile makes everything better and that's really all that matters at the end of the day.
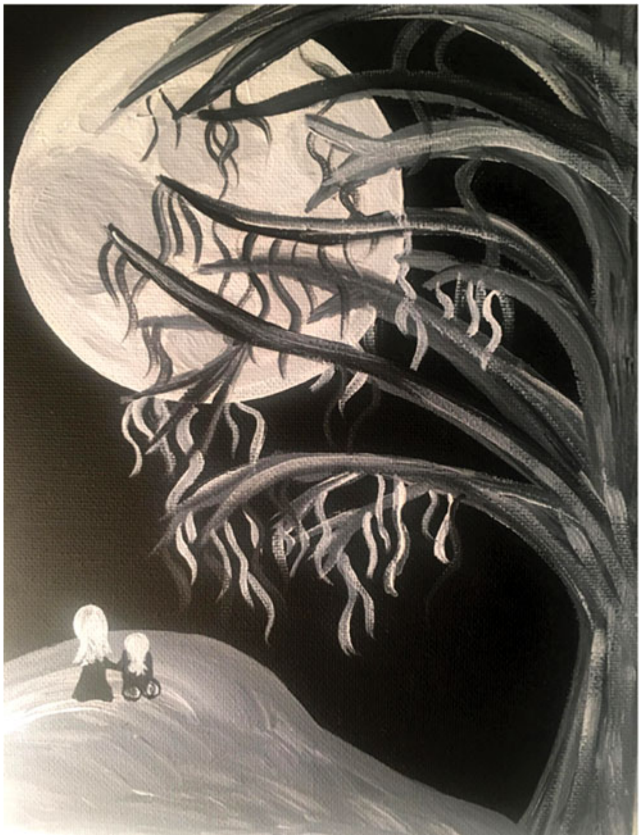	This is another painting I did that represents how I felt after I became a caregiver. It's me and my daughter, staring at the moon together, holding hands. And she brought life back into me.

(*continued*)

Supplementary Table S1. (Continued)

Photograph	Complete narrative
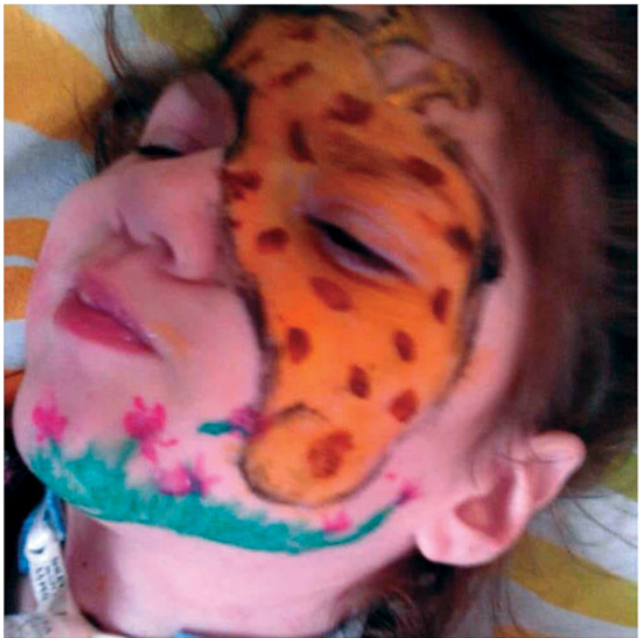	Just because you have a life-limiting illness doesn't mean you have to live every day like your life is limited. We spent most of our days making the best of it. Between therapy appointments and doctor's visits, we always took time to be creative and have fun. We'd laugh and play and read and sing and color and even face paint. Some days were great; others were not. Either way, we always made the best of it.
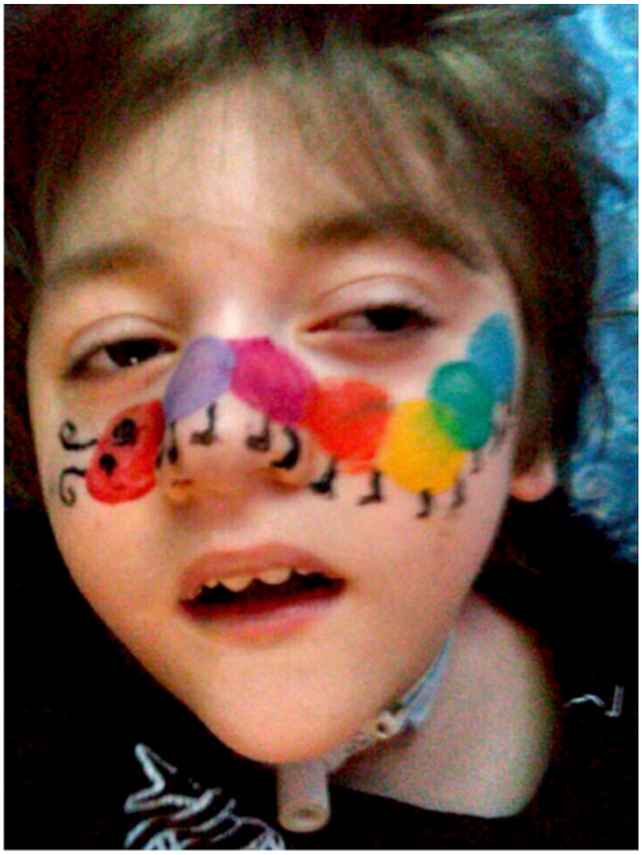	When I say we're making the best of it, I genuinely mean it. Something to think about with families like ours is that we have spent at least one of every holiday in the hospital with one of these children. And I'm talking New Year's and Valentine's Day and Easter and Memorial Day and Fourth of July and Labor Day and Halloween and Thanksgiving and Christmas and birthdays and our own birthdays and their own birthdays and our sibling's birthdays and grandma and grandpa's birthdays. And as much as it sucks, we continue to make the best of it. So when you're at home with your families on these holidays and you're enjoying your time, please think about the families who are in the hospital-also enjoying their time, but in a very different way.

[a]Please note that at the community photograph exhibit, QR codes allowed people to engage in the photovoice narrative directly and hear the voices of the PPCGs talk about their post. Furthermore, as noted in the article, all participants signed a media waiver.

POM-PPCG, Photographs of Meaning Program for pediatric palliative caregiver.
